# The Benefit of a Human Bone Marrow Stem Cells Concentrate in addition to an Inorganic Scaffold for Bone Regeneration: An *In Vitro* Study

**DOI:** 10.1155/2015/240698

**Published:** 2015-01-22

**Authors:** J. Torres, A. Lopes, M. A. Lopes, M. Gutierres, A. T. Cabral, M. H. Fernandes, E. Monteiro, C. F. van Eck, J. D. Santos

**Affiliations:** ^1^Faculdade de Medicina da Universidade do Porto (FMUP), Hospital de São João, Largo Hernâni Monteiro, 4200 Porto, Portugal; ^2^CEMUC, Departamento de Engenharia Metalúrgica e Materiais, Faculdade de Engenharia, Universidade do Porto, Rua Dr. Roberto Frias, 4200-465 Porto, Portugal; ^3^Faculdade de Medicina Dentária da Universidade do Porto (FMDUP), Rua Dr. Manuel Pereira da Silva, 4200-393 Porto, Portugal; ^4^University of Pittsburgh Medical Center (UPMC), Kaufman Building, Suite 1011, Pittsburgh, PA 15213, USA

## Abstract

*Background*. This work compares the osteoblastic behaviour of a bone marrow (BM) aspirate and a prepared BM concentrate of nucleated cells associated with a glass reinforced hydroxyapatite composite (GRHC) in a microporous pellet formulation. *Methods*. BM aspirate (30 mL) was collected during 3 orthopedic surgical procedures, and a concentration system was used to achieve 3 rapid preparations of a concentrate of nucleated cells (3 mL) from the BM aspirates. The BM aspirates (53% cell viability; 2.7 × 10^6^ nucleated cell/mL) and the BM concentrates (76% cell viability; 2 × 10^7^ nucleated cell/mL) were cultured over glass reinforced hydroxyapatite pellets, at the same volume/mass ratio, for 30 days. Cultures performed in standard tissue culture plates were used as control. *Results*. The colonized BM concentrate/material constructs exhibited a representative osteoblastic proliferation/differentiation pathway, evidenced by a high alkaline phosphatase (ALP) activity, expression of collagen type 1, ALP, BMP-2, M-CSF, RANKL, and OPG, and formation of a calcium phosphate mineralized matrix. A clear improved behaviour was noticed compared to the BM aspirate/material constructs. *Conclusions*. The results suggest the benefit of using an autologous BM concentrate/material construct in the clinical setting, in bone regeneration applications.

## 1. Introduction

The availability of bone tissue is one of the greatest concerns of the orthopaedic surgeon, when performing surgery, especially, when dealing with bone defects as the ones present in revision arthroplasty procedures, tumors or fractures [1], situations in which the availability of bone tissue is crucial for a good clinical outcome. It is undeniable that the best bone substitute is bone itself. It is osteoconductive [2], and it has osteogenicity and osteoinductivity due to the presence of stem cells and growth factors, respectively [2]. In addition, it responds to mechanical stimuli, with the production of new bone [3, 4]. Despite all these qualities, autologous bone graft is associated with nonnegligible donor site morbidity [5] and the amount available for use is limited. Allograft is another possible choice [6], but besides the inherent risks of disease propagation [7], its biochemical and mechanical properties are clearly inferior to autograft [8]. These facts make the quest for bone substitutes especially important. Ideally, a bone substitute should have all the qualities of autologous bone graft, without its problems or limitations. The characteristics described in the Diamond Concept—osteoconductivity, osteogenicity, osteoinductivity, and mechanical stimulus [3, 4]—should direct this quest.

This study focusses on the* in vitro* osteogenic behavior of a bone marrow aspirate and a prepared bone marrow concentrate of nucleated cells, along with a modified synthetic hydroxyapatite scaffold [9], in a recently developed microporous pellet formulation [10]. Preliminary* in vivo* studies in a sheep model showed promising results of this scaffold in the regeneration of bone defects [11]. This study aimed to obtain experimental data that support the* in vivo* use of such a cell/material construct. It was hypothesized that a bone marrow concentrate of nucleated cells (which contains osteogenic stem cells and osteoinductive factors), associated with an improved formulation of an inorganic scaffold (a osteoconductive material), would show to be a viable regenerative system.

## 2. Materials and Methods

### 2.1. GRHC Pellets

The used glass-reinforced hydroxyapatite is composed of modified calcium phosphates with controlled percentages of ionic species, such as magnesium, sodium, and fluoride, much like the chemical composition of the mineral phase of human bone [9–13].

The presence of *α*- and *β*-tricalcium phosphate (TCP) secondary phases in its composition affords a higher solubility than HA^14^ and contributes to the release of the referred ionic species. Additionally, the presence of a vitreous liquid phase during the sintering process allows the formation and homogeneous dispersion of *α*- and *β*-TCP phases in the HA matrix. As these phases are biodegraded more rapidly than the matrix, this dispersion assures a homogeneous degradation of the scaffold and, therefore, prevents a too rapid release of microparticles from the material, which could become targets for macrophage phagocytosis [9–13]. Previous reports from the authors revealed an improved* in vitro* performance and an excellent* in vivo* osteointegration with a sustained controlled resorption of the material [14, 15].

This study was performed using pellets obtained through a patented process [10], which requires the use of techniques such as extrusion and spheronization after mixing of hydroxyapatite and a bioglass (with the composition 65P_2_O_5_-15CaO-10CaF_2_-10Na_2_O, mol%) with microcrystalline cellulose. Pellets were then submitted to a thermal treatment [10]. Before bone marrow cell seeding, GRHC pellets were sterilized by autoclaving (120°C, 20 min).

Detailed physicochemical profile of GRHC pellets was recently reported [11]. The pellet formulation presents a particle size range of 1000–4000 *μ*m, surface rugosity and, also, microposity, which favors ions exchanges, cell attachment, and migration through the scaffold.

### 2.2. Preparation of the Bone Marrow Concentrate of Nucleated Cells

Human bone marrow was collected during 3 orthopedic surgical procedures, from the posterior iliac crest of a 65-year-old and a 59-year-old male patient and a 62-year-old female patient, with patient informed consent. The authors chose older patients in order to best mimic the population which usually suffers more from fractures/delayed unions and have less dense bone.

Bone marrow (BM) was extracted with a needle coated with heparin. After the extraction of the bone marrow aspirate (30 mL), the cell separator was loaded with the aspirate and centrifuged at 3200 rpm for 15 min. After removing the plasma from the cell separator, the portion of nucleated cell concentrate was extracted (3 mL). For this operation, a commercially available bone marrow concentration system (BMCS) was used (Biomet Marrowstim).

Flow cytometry was performed to analyze the bone marrow aspirate and the bone marrow concentrate for cell viability and number of total nucleated cells and hematopoietic CD34+ cells (Stem-Kit; Beckman Coulter, Ref IM3630).

### 2.3. Colonization of the GRHC Pellets

Bone marrow concentrate (3 mL) and bone marrow aspirate (3 mL) were diluted with culture medium (5 mL) and were seeded over GRHC pellets placed in 24-well plates, at the same volume/mass ratio, corresponding to a concentration of, respectively, 3 × 10^6^ and 5 × 10^5^ nucleated cells over 0.10 g/cm^2^ of GRHC pellets (mean values). Cultures performed in standard tissue culture plates (absence of materials) were used as control.

Cultures—seeded GRHC pellets and control—were performed in *α*-MEM containing 15% fetal bovine serum (FBS), 100 IU/mL penicillin, 2.5 *μ*g/mL streptomycin, and 2.5 *μ*g/mL amphotericin B and were supplemented with 50 *μ*g/mL ascorbic acid and 10 mM of *β*-glycerophosphate. Incubation was carried out in a humidified atmosphere of 95% air and 5% CO_2_ at 37°C. Culture medium was changed twice a week. Cultures were maintained for 30 days and characterized throughout the culture time for viability/proliferation and osteoblastic differentiation.

#### 2.3.1. MTT Assay

Cell viability/proliferation of the seeded GRHC samples and control cultures was determined by the MTT assay that relies on the ability of viable cells to reduce MTT (3-(4,5-dimethylthiazol-2-yl)-2,5-diphenyltetrazolium bromide) to an insoluble purple formazan product. Cultures were incubated with 0.5 mg/mL of MTT for the last 4 h of the culture period tested. Then, the materials were transferred to a new plate, the formed formazan salt was dissolved with dimethyl sulfoxide, and the absorbance was determined at 600 nm. MTT assay was performed at days 1, 4, 9, 17, 23, and 30.

#### 2.3.2. Total Protein and Alkaline Phosphatase Activity

Total protein content of colonized GRHC and control cultures was assessed in 0.1 M NaOH cell lysates according to the method of Lowry using bovine serum albumin as standard.Alkaline phosphatase (ALP) activity was assayed in cell lysates (obtained by the treatment of the colonized material and control cultures with 0.1% triton), by hydrolyses of *p*-nitrophenol phosphate in alkaline buffer solution, pH 10.3, for 30 min, and colorimetric determination of *p*-nitrophenol at 405 nm. Both ALP and total protein content assays were performed at days 9, 17, 23, and 30. ALP activity was normalized to total protein content and was expressed in nanomoles of *p*-nitrophenol produced per min per *μ*g of protein (nmolmin^−1^/*μ*gprotein).

#### 2.3.3. Gene Expression by Reverse-Transcription Polymerase Chain Reaction (RT-PCR)

Cultures established with the BM concentrate, both control cultures and colonized GRHC pellets, were assessed at day 17 for the expression of the housekeeping gene GAPDH (glyceraldehyde-3-phosphate dehydrogenase) and the osteoblastic genes Collagen 1 (Col 1), ALP, bone morphogenetic protein-2 (BMP-2), osteoprotegerin (OPG), monocyte-colony stimulation factor (M-CSF), and receptor activator of nuclear factor-*κ*B ligand (RANKL). Cultures established with the BM aspirate did not yield sufficient mRNA to perform RT-PCR analysis.

Total RNA was extracted using the RiboPure Kit according to the manufacturer's instructions. The concentration and purity of total RNA in each sample were determined by UV spectrophotometry at 260 nm and by calculating the *A*_260 nm_/*A*_280 nm_ ratio, respectively. RNA, 0.5 *μ*g, was reversely transcribed and amplified (25 cycles) with the Titan One Tube RT-PCR System (Roche), with an annealing temperature of 55°C. The primers used are listed on Table 1. After analysis on a 1% (w/V) agarose gel, the RT-PCR products were densitometric analysed with ImageJ 1.41 software and normalized for the corresponding GAPDH value of each experimental condition.

#### 2.3.4. Scanning Electron Microscopy (SEM)

For SEM observation, cell cultures were fixed (1.5% glutaraldehyde in 0.14 M sodium cacodylate, 10 min), dehydrated in graded series of alcohols and further dried with hexamethyldisilazane. Samples were mounted onto aluminium supports, super-coated with gold, and observed in a Joel JSM 35C scanning electron microscope equipped with an X-ray energy dispersive spectroscopy voyager XRMA System, Noran Instruments.

### 2.4. Statistical Analysis

Three replicas were performed for each assay. Results of MTT and ALP activity are presented as mean ± standard deviation (SD). Comparisons between seeded GRHC samples and the control were performed by the Student's *t*-test. Differences were considered statistically significant at *P* < 0.05.

### 2.5. Ethics Statement

All patients were completely informed about the risks of the procedure of bone marrow collecting that was done during the different orthopaedic procedure they were admitted to. They were also informed about the purpose and management of the future use in the laboratory of their bone marrow aspirate and that it would be destroyed at the end of the experiments.

All patients signed informed consent. This study and the informed consent were reviewed and approved by the Ethics Committee of the Institution (C.E.S.-H.S.J.).

## 3. Results

**Figure 1:**
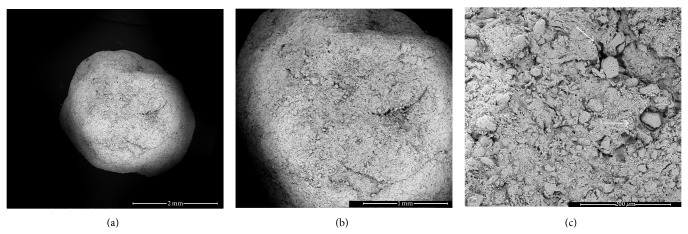
Representative SEM images of GRHC pellets. (a) and (b) pellets with irregular details on the surface; (c) microporosity on the pellets surface (arrows). (a) Bar, 2 mm; (b) 1 mm; (c) Bar, 200 *μ*m.

Figure 1 shows representative SEM images of GRHC pellets. Results showed that GRHC pellets presented a spherical shape with a particle size range of 1000–4000 *μ*m, a microporosity of 25.3%, and a surface area of 0.0171 m^2^/g. Pellets exhibited surface rugosity and, also, microposity, which increases surface area.

Flow cytometry analysis (Table 2) shows the differences in cell viability, concentration of nucleated cells, concentration of CD34+ cells, and expected percentage of mesenchymal stem cells (MSCs) [16–18], between the BM concentrate and aspirate.

**Figure 2:**
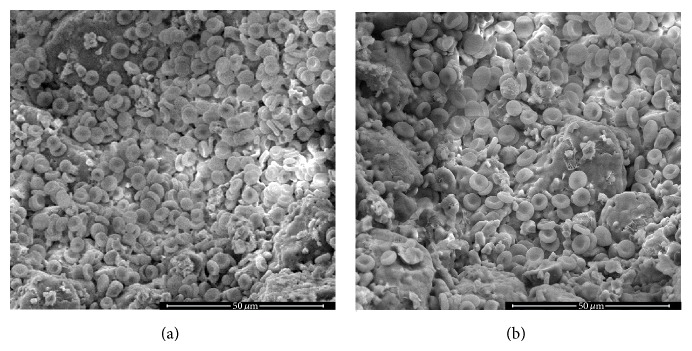
Representative SEM images of GRHC pellets seeded with bone marrow aspirate (a) and bone marrow concentrate (b), at 1 day of culture. The bone marrow suspension contained erythrocytes which were removed during subsequent medium changes. Bar: 50 *μ*m.

Figure 2 shows SEM observation of GRHC pellets seeded with the BM aspirate or the BM concentrate 1 day after plating.

**Figure 3:**
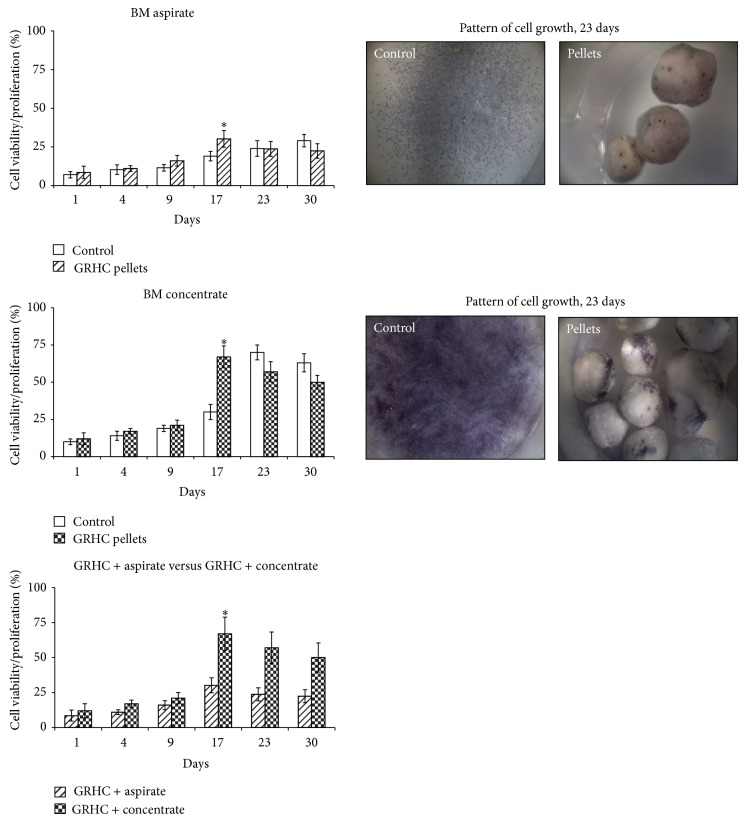
MTT assay. Cell viability/proliferation of cultures established with BM aspirate or BM concentrate performed over standard tissue culture plates (control) and GRHC pellets (maintained for 30 days), and direct comparison between both constructs. ^*^Significantly different. The pattern of cell growth observed in both conditions is also shown in 23-day cultures (cultures were treated with MTT for 4 hours, and viable/proliferating cells reduced the MTT to a purple formazan reaction product); 20x.

Figure 3 (MTT assay), compares the MSCs viability/proliferation in the BM aspirate, the BM concentrate and control cultures (grown on standard tissue culture plates). Figure 3 also shows the pattern of cell growth via the formation of the purple formazan product in the MTT assay, before the dye dissolution, in 23 day-cultures, providing a general view of the pattern of cell growth and spatial distribution of the adherent MSCs present in the BM aspirate and the BM concentrate, compared to control cultures.

**Figure 4:**
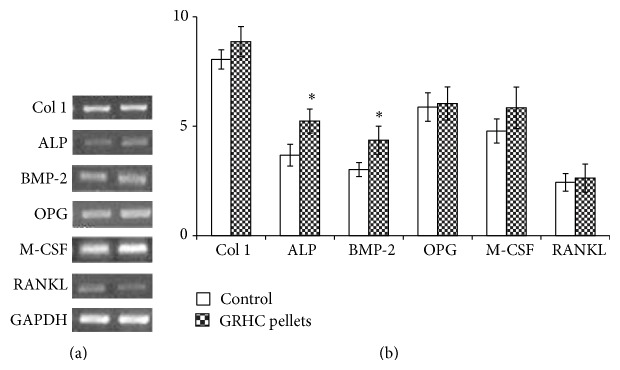
Gene expression profile of cultures established with BM concentrate, performed on standard tissue culture plates (control) and GRHC pellets, at day 17; (a) representative agarose gel of the PCR products; (b) densitometric analyses of the PCR products, normalized to the corresponding GAPDH value.

Expression of osteoblastic genes can be seen on Figure 4, through PCR data on GRHC pellets colonized with BM concentrate and controls, assessed at day 17, with expression of Col 1, ALP, BMP-2, OPG, M-CSF, and RANKL. In the same experimental conditions (17-day culture time), cultures performed with the BM aspirate did not yield sufficient amount of RNA to perform PCR analysis, due to the significantly lower number of cells found in this situation.

**Figure 5:**
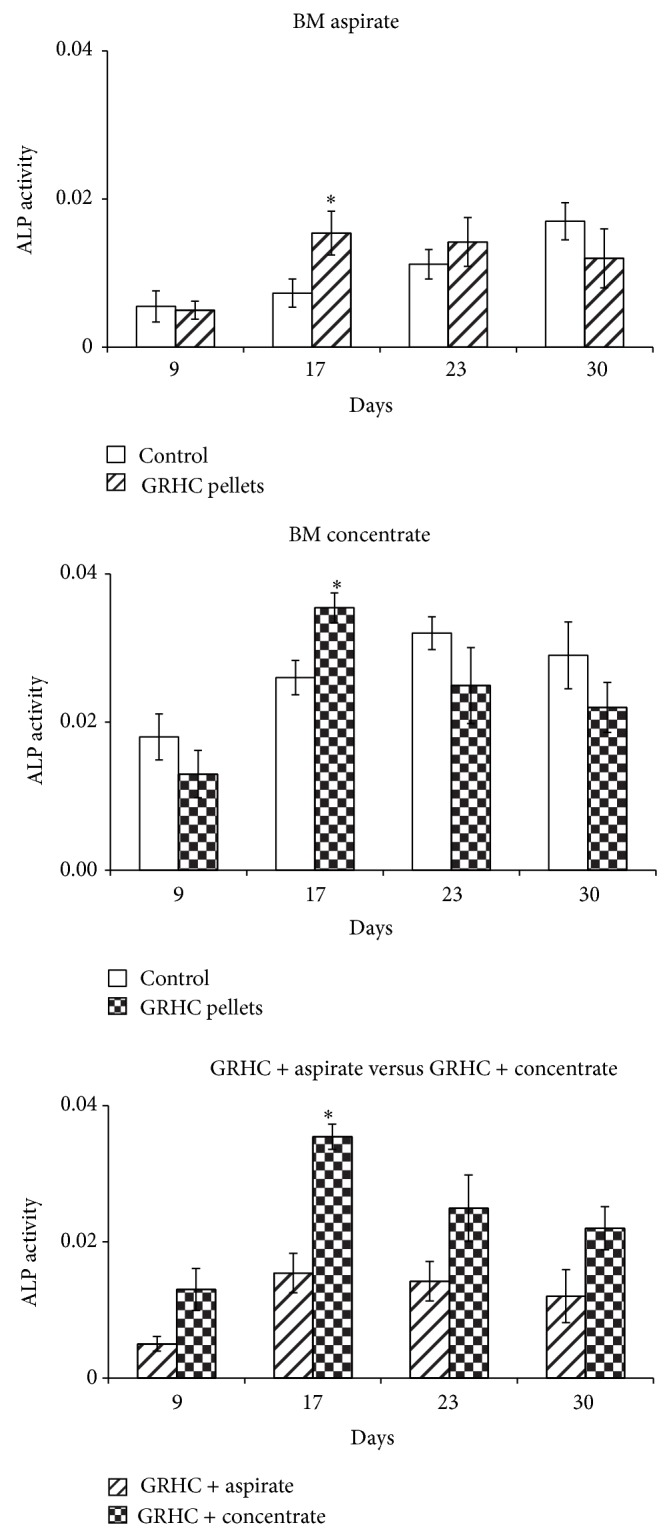
ALP activity of cultures established with BM aspirate or BM concentrate performed over standard tissue culture plates (control) and GRHC pellets (maintained for 30 days) and direct comparison between both constructs. ^*^Significantly different.

Figure 5 shows ALP activity of control (aspirate and concentrate) and pellets cultures (aspirate and concentrate).

**Figure 6:**
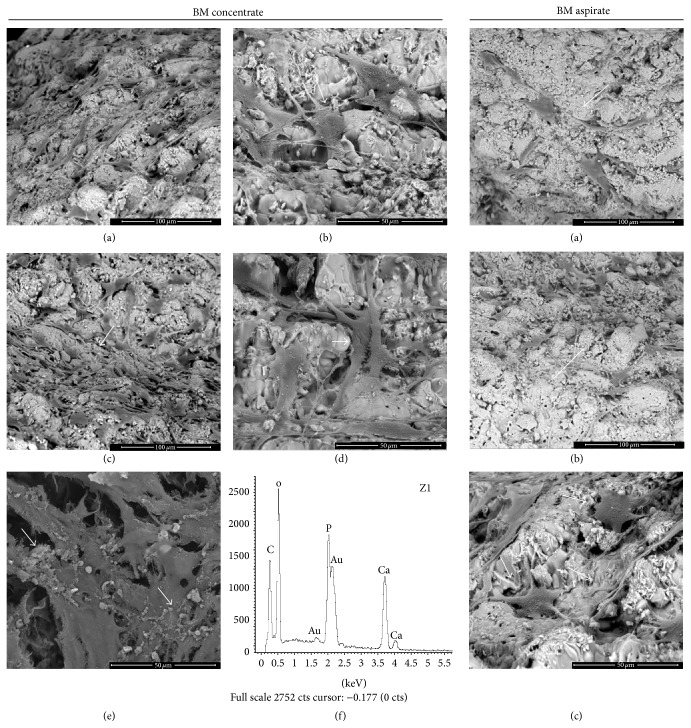
Representative SEM images of GRHC pellets colonized with BM concentrate or BM aspirate, at 17 and 23 days. Pellets colonized with BM concentrate at 17 ((a) and (b)) and 23 ((c)–(e)) days; (a) and (c) clusters of cells on the material surface; (b) and (d) high magnification images showing the cells taking advantage of the microporosity to attach to the material surface (arrows); (e) formation of globular mineral deposits closely associated with the cell layer (arrows); (f) X-ray spectrum of the mineral deposits showing Ca and P peaks. (a) and (c) Bar, 100 *μ*m; (b), (d), and (e) Bar, 50 *μ*m. Pellets colonized with BM aspirate at 17 (a) and 23 ((b) and (c)) days; (a) and (b) clusters of cells (arrows) on the material surface; (c) high magnification image showing the cells attached to the microporous of the material surface (arrow). (a) and (b) Bar, 100 *μ*m; (c) Bar, 50 *μ*m.

Figure 6 contains SEM images of GRHC pellets colonized with BM aspirate and concentrate (at day 17 and 23), as well as X-ray spectrum analyses of numerous globular mineralized deposits (Ca and P peaks)

## 4. Discussion

In this work, human BM aspirates and prepared BM concentrates of nucleated cells were seeded over a glass reinforced Hydroxyapatite^18^ in a microporous pellet formulation [10]. The main purpose was to compare the osteoblastic proliferation and differentiation evolution of the two BM suspensions seeded over the GRHC pellets, aiming to achieve laboratory data that can support the benefit of using a BM concentrate/material construct in the clinical setting, in bone regeneration applications, such as bone defects present in revision arthroplasty procedures, tumors, or fractures. The data obtained clearly shows the benefit of using this construct; it provides both the inorganic and the biological properties necessary for bone growth, in an accelerated way, allowing for an almost endless list of possible new applications.

### 4.1. The Inorganic Scaffold Selected

In the present study, GRHC pellets were tested as an improved recent formulation, prepared through an extrusion and spheronization process [9]. Results showed that GRHC pellets presented a spherical shape with a particle size range of 1000–4000 *μ*m, a microporosity of 25.3%, and a surface area of 0.0171 m^2^/g. Pellets exhibited surface rugosity and, also, microposity, which increases surface area, and it is expected to favor cell adhesion. These characteristics are important for the formation of interconnecting pores between the pellets witch, according to some authors [19], are of critical importance for osseous ingrowth.

### 4.2. The Bone Marrow Aspirate and the Bone Marrow Concentrate

In this work, BM concentrate of nucleated cells was prepared from BM aspirates collected during 3 orthopedic surgical procedures, using a BMCS. The same volume/mass ratio was used to prepare the bone marrow/material constructs. This is because, in the clinical setting, this ratio would be the same, either using a BM concentrate or a BM aspirate, as it corresponds to an optimized volume of bone marrow and amount of material to achieve the proper consistency of the cell/material construct to fill a specific bone defect.

Flow cytometry analysis (Table 2) showed that BM concentrate presented a significantly higher mean concentration of nucleated cells (2 × 10^7^ cell/mL) compared to that on BM aspirate (2.7 × 10^6^ cell/mL), which is expected considering the experimental protocol, as 30 mL of BM aspirate yielded 3 mL of BM concentrate. Of these, the mean number of hematopoietic CD34+ was 8 × 10^4^ cell/mL and 8 × 10^3^ cell/mL, respectively, in the BM concentrate and the BM aspirate. According to previous studies reporting that the percentage of mesenchymal stem cells (MSCs) in the nucleated cell fraction harvested from the BM is 0.01 to 0.001% [16–18], the BM concentrate contained a higher number of MSCs, about 10 times of that expected in the BM aspirate. In addition, flow cytometry analysis (Table 2) also showed that mean cell viability was found to be higher in the BM concentrate (76%) compared to that on BM aspirate (53%), most probably because the process of BM concentration has eliminated a number of nonviable cells. As the same volume/mass rate was used to prepare the cell/material constructs, a better performance is anticipated with the BM concentrate/GRHC pellets regarding the extent of material colonization.

### 4.3. Performance of the Bone Marrow/GRHC Pellets Constructs

SEM observation of GRHC pellets seeded with BM aspirate or BM concentrate 1 day after plating (Figure 2), showed the way cells easily adhere to the surface of pellets.

#### 4.3.1. Cell Viability/Proliferation: Pattern of Cell Growth

Figure 3, regarding the MTT assay, evidenced the significant difference in the mean number of MSCs present in the BM aspirate and the BM concentrate. Control cultures (grown on standard tissue culture plates) performed with the BM aspirate presented a low growth rate, showing a slow increase in the cell viability/proliferation until the end of the culture time (this growth rate was not statistically significant in comparison to the GRHC pellets, except for day 17). However, cultures established with the BM concentrate exhibited higher mean MTT reduction values at all tested time-points and also a higher growth rate, especially from days 17 to 23; maximum values were achieved by day 23 and, after that, cell proliferation decreased slowly. Also, GRHC pellets colonized with BM concentrate yielded a significantly higher mean cell viability/proliferation compared to those seeded with BM aspirate. It is worth to note that, compared to control cultures, maximum values were achieved earlier in the colonized material samples (either with the BM concentrate or the BM aspirate), that is, around day 17.

Figure 3 also shows the formation of the purple formazan product in the MTT assay, before the dye dissolution, in 23-day cultures, providing a general view of the pattern of cell growth and spatial distribution of the adherent MSCs present in the BM aspirate and the BM concentrate. Control cultures exhibited a uniform cellular distribution, but the cultures established from the BM concentrate presented a significantly higher cell density and the presence of cellular groups over the culture plate. On the GRHC pellets, MSCs used the topographic irregularities on the surface to adhere and proliferated with culture time within specific surface locations forming well-defined cell clusters scattered over the material surface. The number and extension of the cellular clusters were significantly higher over the pellets seeded with the BM concentrate. In addition, spreading of the colonized area over the material surface was evident.

#### 4.3.2. Expression of Osteoblastic Genes

GRHC pellets colonized with BM concentrate, assessed at day 17 (culture time exhibiting the highest cell viability/proliferation), expressed Col 1, ALP, BMP-2, OPG, M-CSF, and RANKL (Figure 4). In addition, increased expression levels were found for ALP and BMP-2, compared to cultures performed on standard tissue culture plates.

Collagen type 1 is the most abundant extracellular bone matrix, being considered an early bone differentiation marker, which has a role in osteoblastic differentiation and also in the nucleation site and growth space of hydroxyapatite [20]. Also, ALP gene expression represents a frequently used early marker for osteogenic differentiation, as ALP has a determinant role in the mineralization of the extracellular collagenous matrix, by providing phosphate ions that, with calcium ions, are used in the formation of the cell-mediated calcium phosphate mineralized matrix [20]. BMPs are members of the TGF-beta superfamily of cytokines that affect bone formation by inducing osteoblast differentiation and maintenance of mature osteoblasts [21]. Cultures also expressed M-CSF, RANKL, and OPG. This is a relevant issue, as osteoblastic cells, in addition to being responsible for the bone formation events, are essential modulators of the osteoclastogenesis, through the production of a variety of molecules [22]. Among them, M-CSF and RANKL, acting on RANK and c-Fms receptors on osteoclast cells, respectively, are necessary for osteoclast survival, differentiation, and activation [23]. On the other hand, OPG is a decoy receptor that binds to RANKL, blocking RANKL from binding to the RANK receptor on osteoclasts, inhibiting osteoclastogenesis [24]. Therefore, these results suggest that osteoblastic cells arising from BM concentrate exhibit a normal behavior regarding paracrine pathways of osteoblast/osteoclast communication, which are essential for bone development and remodeling.

In the same experimental conditions (17-day culture time), cultures performed with the BM aspirate did not yield sufficient amount of RNA to perform PCR analysis, due to the significantly lower number of cells found in this situation.

#### 4.3.3. Functional Parameters: ALP Activity and Matrix Mineralization

Control cultures established with BM aspirate presented low ALP activity, which increased gradually during the 30-day culture period. However, cultures performed with BM concentrate showed significantly higher values, and maximal activity was attained at days 23–30. Cultures growing over the GRHC pellets displayed a similar pattern, that is, with higher values being seen in the material colonized with the BM concentrate. A significant increase was observed between days 9 and 23, suggesting an osteoblastic differentiation pathway [20]. Compared to control cultures, colonized pellets exhibited earlier maximal ALP activity, around day 17. Results are shown in Figure 5.

#### 4.3.4. SEM and X-Ray Spectrum Evaluation

SEM images of GRHC pellets colonized with BM concentrate are shown in Figure 6. MSCs were able to attach and spread over the material surface taking advantage of the surface microporosity and topography. Cells proliferated with culture time, and, by days 17 and 23, abundant cell clusters were visible over the material surface. These clusters appeared to be associated with particular surface features, namely, small defects scattered over the surface, where it was easier to create cellular niches. Within the cell clusters/niches, cell layer appeared well organized with established cell-to-cell communication, abundant cytoplasmic extensions, and a perfect adaptation to the underlying material microporosity and topography. In addition, by day 23, the cell clusters presented numerous globular mineralized deposits in close association with the cell layer, which showed the presence of Ca and P peaks on X-ray analyses. These localized areas of cell growth progressively propagated to the neighboring surface, in an attempt to colonize the entire pellet.

Comparatively, on the GRHC pellets colonized with BM aspirate, cell growth was limited to small niches (Figure 6), and evidence of matrix mineralization was not observed at 23-day culture time, which might be related with the low cell growth rate and ALP activity observed in these conditions. This observation suggests the need for a longer culture time to achieve the appropriate osteoblastic proliferation/differentiation behaviour, because of the low number of MSCs present in the BM aspirate compared to that in the BM concentrate.

### 4.4. Performance of the GRHC Pellets

Regarding the performance of the selected material scaffold, GRHC pellets colonized with BM concentrate or BM aspirate showed maximum values for cell viability/proliferation and ALP activity at an earlier culture time, compared to that seen in control cultures grown in standard tissue culture plates. In addition, increased ALP activity and higher expression of ALP and BMP-2 genes were found on the pellets colonized with BM concentrate. This improved osteoblastic performance of GRHC pellets, compared to the standard polystyrene surface, is in agreement with previous* in vitro* studies involving GRHC granules [15]. As mentioned above, GRHC is composed of an HA matrix with bioresorbable *β*- and *α*-TCP phases, which are more soluble than single HA and liberate Ca and P ionic species to the local environment. Surface reactions occurring as a result of ongoing dissolution/deposition events contribute to a rapid formation of an apatite layer which appears to induce osteoblastic growth and differentiation [15]. In addition, the presence of fluoride ions in the composition of GRHC may also have a positive contribution, as this ion is known to stimulate osteoblastic cell proliferation [24]. This physicochemical profile apparently contributes to the good performance of this glass-reinforced hydroxyapatite in bone tissue applications.

## 5. Conclusion

The preparation of a BM concentrate from a BM aspirate using a BMCS is an easy and rapid procedure which provides a mononuclear cell suspension rich in MSCs that readily adhere to the microporous surface of a modified synthetic hydroxyapatite scaffold. In addition, the colonized scaffold exhibits a representative osteoblastic proliferation/differentiation pathway, ending up with the formation of a mineralized extracellular matrix. A clear improved behaviour was noticed compared to a similar cell/material construct performed with a BM aspirate, apparently because of the relatively low number of MSCs in this cell suspension. The enrichment of MSCs in a small volume is of utmost importance, considering the low percentage of MSCs in the BM aspirate. These results suggest that the association of autologous BM concentrate of osteoblastic precursor cells with an appropriate scaffold appears to be a promising approach when considering tissue engineering techniques for the management of several clinical problems, such as late unions, fractures, tumors, osteotomies, and revision joint replacement surgery, among others. In these situations, especially in older patients, the availability of bone tissue is of the utmost importance to achieve a good clinical outcome, and, therefore, the proposed BM concentrate/scaffold construct might be a potential solution.

## Figures and Tables

**Table 1:** Primers used on RT-PCR analysis.

Gene	5′ Primer	3′ Primer
GADPH	5′-CAGGACCAGGTTCACCAACAAGT-3′	5′-GTGGCAGTGATGGCATGGACTGT-3′
Col 1	5′-TCCGGCTCCTGCTCCTCTTA-3′	5′-ACCAGCAGGACCAGCATCTC-3′
ALP	5′-ACGTGGCTAAGAATGTCATC-3′	5′-CTGGTAGGCGATGTCCTTA-3′
BMP-2	5′-GCAATGGCCTTATCTGTGAC-3′	5′-GCAATGGCCTTATCTGTGAC-3′
OPG	5′-AAGGAGCTGCAGTACGTCAA-3′	5′-CTGCTCGAAGGTGAGGTTAG-3′
M-CSF	5′-CCTGCTGTTGTTGGTCTGTC-3′	5′-GGTACAGGCAGTTGCAATCA-3′
RANKL	5′-GAGCGCAGATGGATCCTAAT-3′	5′-TCCTCTCCAGACCGTAACTT-3′

**Table 2:** Flow cytometry analyses of Bone marrow concentrate and Bone marrow aspirate (mean values 3 donors ±SD).

	Cell viability	Total of nucleated cells	CD34+ cells	Expected Mesenchymal Stem Cells [25]
BM concentrate	76% (±4%)	2.0 × 10^7^ cell/mL (±6.0 × 10^6^)	8.3 × 10^4^ cell/mL (±2.5 × 10^4^)	200–2000 cell/mL
BM Aspirate	53% (±5%)	2.7 × 10^6^ cell/mL (±1.1 × 10^6^)	8.0 × 10^3^ cell/mL (±3.2 × 10^3^)	27–270 cell/mL
